# The Vienna self-assessment questionnaire: a usable tool towards more health-literate hospitals? Explorative case studies in three hospitals in Belgium

**DOI:** 10.1186/s12913-021-06211-y

**Published:** 2021-03-31

**Authors:** Gilles Henrard, Marc Vanmeerbeek, Nadia Dardenne, Jany Rademakers

**Affiliations:** 1 Department of General Practice/Family Medicine; Research Unit Primary Care & Health, University of Liège, 13 avenue Hippocrate, Quartier Hôpital B23, 4000 Liège, Belgium; 2 Department of Public Health, Biostatistics unit, University of Liège, Liège, Belgium; 3 Nivel, PO box 1568, 3500 BN Utrecht, the Netherlands; 4 Department of Family Medicine, CAPHRI, Maastricht University, PO Box 616, 6200 MD Maastricht, the Netherlands

**Keywords:** Health literacy, Accessibility of health services, Organisational culture, Process assessment (health care), Organisational innovation, Quality improvement, Organisational health literacy, Health literate organisation, Health literacy responsiveness, self-assessment

## Abstract

**Background:**

Strengthening the capacity of hospitals to take into account the level of health literacy of their public is a necessity to improve the quality of care. One way to develop adequate health literacy responsive policy and strategies in hospitals is the use of self-assessment tools to raise awareness, help prioritise action and mobilise stakeholders. The Vienna Health Literate Organisation (V-HLO) questionnaire, recently translated and adapted into French, is designed to meet this objective. In this study we have piloted the French version of the V-HLO (V-HLO-fr) tool in the main hospitals of Liège (Belgium) to explore its feasibility and gain a first insight into the strengths and weaknesses of the health literacy responsiveness of the participating hospitals.

**Methods:**

We performed explorative case studies in three hospitals. Our mode of application of the V-HLO-fr was inspired by the ‘RAND Appropriateness’ method: first, individual members of an internal multidisciplinary panel filled out the questionnaire and then the results were discussed collectively in each hospital during a ‘round table’ meeting. The feasibility of the process was assessed by direct observation of the round tables and with semi-structured phone interviews.

**Results:**

The V-HLO-fr tool was fully applied in the three targeted hospitals and the process seems to be acceptable, practicable and integrable. Strengths (e.g. the facilitation of patient navigation to the hospital) and weaknesses (e.g. the provision of easy to read, understand and act on health information materials) in terms of health literacy responsiveness have been highlighted.

**Conclusion:**

V-HLO-fr can be a suitable tool for a needs assessment that allows hospitals to create awareness and formulate targeted actions to further strengthen their health literacy responsiveness. Its mode of application, formalised by taking inspiration from the RAND method, could be further improved by paying more attention to recruiting and supporting participants. The V-HLO-fr and its added value in real-world projects should now be further tested in a larger number of hospitals.

**Supplementary Information:**

The online version contains supplementary material available at 10.1186/s12913-021-06211-y.

## Background

### Health-literate hospitals

Patients with limited health literacy, defined as the capacity to find, understand, appraise and apply health information [1], have more difficulties understanding written and digital information material, navigating the health care system, and communicating with care providers [2]. Their health outcomes and experiences with health care are worse compared to patients with sufficient health literacy [3].

To improve health care delivery, hospitals should become more sensitive to the different needs and skills of patients with limited health literacy. This organisational responsiveness is also referred to as being ‘health literate’, i.e. the organization makes it easier for people to navigate, understand and use information and services to take care of their health. Brach et al. [4] discerned 10 attributes of health literate care organizations^1^.

‘Health-literate hospitals’ provide safer patient care [5], are more open for user participation [6] and better placed to deal with social health inequalities [7]. Working to make hospitals more health literate involves much more than just focussing on strengthening individual capacities of care providers, at the micro level. Structural changes in terms of work culture and material conditions must take place as well [8]. While the importance of policy support, at the macro level, cannot be overlooked [9], each hospital can act at its own meso level.

### Self-assessment tools

A first step towards becoming a health-literate hospital is a diagnosis of the strengths and weaknesses regarding organisational health literacy. Self-assessment tools are a key approach to tackling the problem by raising awareness, helping to prioritise action, and mobilising stakeholders around the issue [10]. In a recent systematic literature review [11], Farmanova et al stated that these tools were generally considered feasible and easy to use. Notably, the authors highlighted the ‘Vienna Health Literate Organisation’ (V-HLO) tool of Dietscher et al, developed in 2015 [12], a questionnaire made up of 9 standards, 22 sub-standards and 160 items (Table 1). It was successfully tested in Austria in 2016 by Pelikan and Dietscher [13]. In the same year an international work group was created within the ‘International Network of Health Promoting Hospitals’^2^ with a view to stimulating translation, adaptation and adoption of the tool.

**Table 1:** The 9 standards and the 22 sub-standards of the Vienna Health Literate Organisation (from Pelikan and Dietscher 2016) [13]

Standards	Sub-standards
The organisation should: 1. Establish management policy and organisational structures for health literacy	The organisation: 1.1 Understands health literacy as an organisational responsibility 1.2 Ensures quality assurance in the field of health literacy
2. Develop materials and services in participation with relevant stakeholders	2.1 Involves patient representatives in the development of materials and services 2.2 Involves staff in the development of materials and services
3 Qualify staff for health-literate communication with patients	3.1 Ensures that staff are trained for health-literate communication in diagnosis, therapy, treatment and care, and discharge preparation 3.2 Ensures that staff are trained for health-literate communication in disease prevention and health promotion
4 Provide a supportive environment – health-literate navigation and access	4.1 Ensures barrier-free contact by internet and telephone 4.2 Provides all information needed for accessing the organisation 4.3 Ensures sufficient orientation support in the entrance area for patients and visitors to easily find their way 4.4 Has an easy-to-follow navigation system and signage 4.5 Ensures that patients and visitors have access to free health information
5 Apply health literacy principles in routine communication with patients	5.1 Face-to-face communication with patients follows health literacy principles 5.2 Written and audio-visual material are designed according to health literacy principles 5.3 The organisation provides resources to guarantee translation support when needed 5.4 Communication in high-risk situations follows health literacy principles
6 Improve the health literacy of patients and their entourage	6.1 Patients (and their entourage) are supported to improve health literacy for disease-related self-management 6.2 Patients (and their entourage) are supported to improve health literacy for healthy lifestyles
7 Improve the health literacy of staff	7.1 Staff are supported to improve the health literacy they need for managing job-related health risks 7.2 Staff are supported to improve health literacy for healthy lifestyles
8 Contribute to health literacy in the region	8.1 Supports health literacy in continuous and integrated care 8.2 Contributes to the development of health literacy in the regional population
9 Share experiences and be a role model	9.1 Supports the dissemination and further development of concepts and practice of health-literate healthcare

### The Belgian situation

The term “health literacy” is still largely unknown in French-speaking Belgium [14] and there is insufficient attention in general to the underlying problems. In Belgium, one third of the adult population has a low health literacy level [15]. A recent report by a Belgian healthcare knowledge centre sets the benchmarks for a national policy and underlines the need to act at the level of healthcare institutions, among other things through the use of self-assessment tools [16]. Because of the successful evaluation of the V-HLO and its international dissemination opportunity, we decided to translate and adapt this tool for the Belgian French-speaking context, what was described in a previous publication [17] to then explore its feasibility in Belgian hospitals.

### Objectives

The primary objective of this study was to pilot the use of the French version of the V-HLO (V-HLO-fr) tool in Belgian hospitals and explore the feasibility of its application. Specifically, we focused on four of the general areas of feasibility described by Bowen et al. [18], namely: Acceptability, Implementation, Practicality and Integration.

The secondary objective was, by carrying out internal ‘organizational diagnosis’, to gain a first insight into the strengths and weaknesses of each of the participating hospitals in terms of health literacy responsiveness.

## Methods

We used the French translated and validated version of the V-HLO (V-HLO-fr) [17]. In our study, standard 7 (‘improving the health literacy of the staff themselves’) is deleted from the questionnaire, which reduces the number of sub-standards to 20 and items to 149. We deleted this standard to take into account the opinions of experts consulted for the validation of the French translation of the questionnaire indicating a lack of relevance in the French-speaking Belgian context [17]. We globally applied the same self-assessment process used in the original feasibility study conducted by Dietscher and Pelikan [13]. In each hospital, a multidisciplinary panel of internal quality and care managers applied the tool in two stages: first, they individually filled out the questionnaire and then they discussed the results collectively during a round table meeting to produce a more shared organisational diagnosis. This meeting was part of the self-assessment process.

### Recruitment of hospitals and participants

The three main hospitals in Liège, Belgium, were proactively solicited for the study by the main author (GH). The top management of the three hospitals had previously agreed to join a federal pilot improvement project for the management of chronic illnesses,^3^ in which our study was mentioned as one of the interventions, which probably helped its adoption by the middle management. Each hospital has an approximate capacity of 1000 beds to cater for a city of 200,000 inhabitants and a hinterland of 500,000 inhabitants. They are well representative of the diversity of the Belgian hospital landscape: a university hospital, a public hospital managed by various public partners with a more underserved population, and a private hospital with a Christian ethos. The three hospitals are only a few kilometres apart and their recruitment zones overlap widely. Their population is generally representative for the Belgian French speaking population. The Belgian health care system itself is characterized by a high level of accessibility [19]. Patients have free choice of health care professionals and health care institutions (e.g. hospitals) [20].

For the multidisciplinary panel, we invited quality and/or care managers in the broadest sense (medical directors, heads of department, quality managers, logistics managers, human resources managers, hospital mediator (ombudsman) and patients’ representatives) to participate. A contact person in each hospital was chosen, whose role was to recruit other members of their institution who belonged to the targeted groups. The target group should represent the institution in its entirety and not just one unit or department.

### Data collection and analysis

**Fig. 1:**
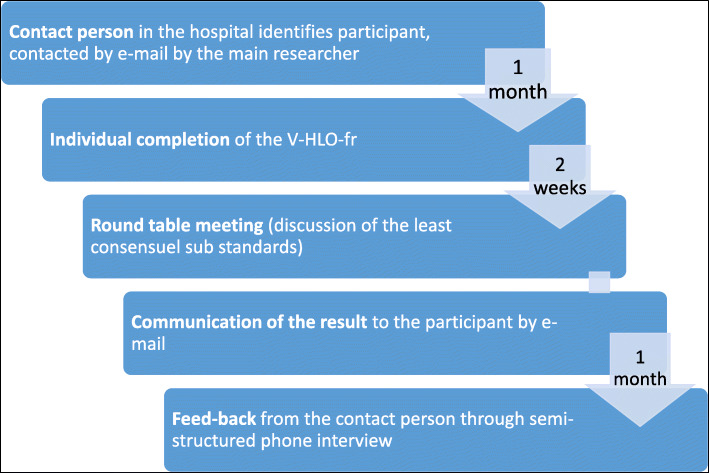
diagram of the case explorative studies

When the panel had been constituted, the members were invited by email to individually complete the V-HLO-fr questionnaire. They were given 1 month to complete the questionnaire, and a reminder e-mail was sent 1 week before the deadline (see Fig. 1).

In order to allow statistical processing of data, a numerical score was attributed to each response category of the questionnaire: 4 for ‘yes’, 3 for ‘rather yes’, 2 for ‘rather no’ and 1 for ‘no’. Each category indicated the degree of fulfilment of the items in the self-assessed entity.

In a second step, 2 weeks after returning their individual questionnaire, the participants were invited to a single, two-hour long, round table meeting in order to discuss the results and produce a more shared diagnosis.

These round table meetings were chaired by the main author of the study (GH) and observed by one of the co-authors (MV). These meetings were structured by a road map (see additional file 1). The discussions took place at the level of the different sub-standards of the questionnaire, dealt with by increasing order of agreement to target the discussions that were most necessary. The discrepancy between respondents for each sub-standard was computed using the ‘average distance between respondents’ (MAE, Mean Absolute Error): the weaker the value, the greater the agreement. The participants were explicitly warned that the objective of the round table was not to reach a consensus at all costs, but to exchange information and opinions allowing for clarification of ‘artefactual disagreements’ due to a different understanding or too partial knowledge of the subject studied.

For each sub-standard addressed during the meeting, the discussion was structured in this way:

1. introduction by the chairman of the scope of the sub-standard under discussion and clarification questions
2. revelation of the mean score of the sub-standard, derived from individual answers given by the participants before the round table meeting
3. spontaneous reaction of participants
4. rating of the sub-standard by each participant using the same scale as for individual rating before the round table meeting. The final score assigned to the sub-standard being the median of the individual scores.

Dietscher and Pelikan [13] identified some limits regarding their application procedure of the questionnaire, especially during the round table meetings. They underlined that hospitals developed different strategies to deal with discrepancies in individual participant rating (e.g. trying to reach consensus, deciding one all ratings with a two-thirds majority or calculating the mean scores for all items) and that this variation could benefit from some further standardisation of the decision process. To address this issue, we drew inspiration from the implementation guide for the ‘RAND Appropriateness’ method of Fitch et al. [21]. The influence of this method took the form of individual quotations of the participants without the necessity for consensus, the presence of a chairman responsible for preparing and facilitating the round tables or the targeting of those discussions that seemed most necessary. Fitch et al. affirm that, fundamentally, the RAND method is a modified Delphi method, enabling the panellists to give their opinions between the quotation turns, and that the biases generated by face-to-face encounters can largely be controlled by a structured animation. The meeting ended at scheduled time with a brief evaluation on the round table experience.

Third, a report of the results was sent to each participant. One month later, a semi-structured telephone interview (see additional file 2) with the contact person was carried out in order to gain a later understanding of the feasibility of the application of the questionnaire and eventual follow-up.

The study was conducted between May 2018 and August 2019. Statistical analyses were carried out using the statistical software programmes SAS version 9.4 and RStudio for Windows.

## Results

### Feasibility of the V-HLO-fr tool in Belgian hospitals

Individual answers were collected and round table meetings held in all three hospitals. In hospital 1, our contact person represented the institution within the patients’ committee in which the project was first briefly presented. She solicited potential participants with the support of the president of the patients’ committee. In hospital 2, the recruitment was led by a head of a nursing department, heavily invested in projects aimed at improving communication with the patients. In hospital 3, the project benefitted from the direct support of a member of the top management, responsible for quality. The profile of the participants is described in Table 2.

**Table 2:** number and functions of the participants in the study (in bold, contact person)

	Participant profile	Participation	
		Questionnaire	Round table
Hospital 1		*n* = 3	*n* = 6
	**Hospital mediator (ombudsman), representative of the institution in the patients’ committee**	x	x
	President of the patients’ committee	x	x
	Head of nursing department	x	x
	Head of department Quality and Institutional Safety		x
	Head of medical department		x
	Head of the logistical services department		x
Hospital 2		*n* = 5	*n* = 8
	**Head of nursing department**	x	x
	Hospital mediator	x	x
	Quality expert	x	x
	Geriatric doctor	x	
	Patient services manager, human resources and communication department	x	
	Coordinator of diversity projects, interpreting		x
	Director of the Strategic Cell, Department, Quality and supervision of strategic projects		x
	Director Quality cell		x
	2 members of the patients’ committee		x
Hospital 3		*n* = 13	*n* = 8
	**Clinical and continued improvement of quality and safety manager**	x	x
	Quality coordinator	x	x
	Head of nursing department	x	x
	Risk manager	x	x
	Assistant director nursing department	x	x
	Pharmacist	x	x
	Assistant manager nursing department	x	x
	Coordinator clinical itinerary	x	x
	Paediatric doctor	x	
	Assistant medical director	x	
	Palliative care doctor	x	
	Assistant human resources director	x	
	Accident and emergency doctor	x	

We present some aspects of the feasibility of the V-HLO-fr derived from our explorative cases studies based on four of the general areas of feasibility described by Bowen et al [18], namely: Acceptability, Implementation, Practicality, and Integration.

1. With regard to **acceptability** (i.e. how the intended individual recipients react to the intervention), the participants had experienced the round tables in a positive way as evidenced by some of the statements from the closing speaking tour.

*“Another place for exchanges”*

*“Reassured about the value of what we do”*

*“Putting into words what we feel”*

2) The feedback received through telephone interviews with the contact person was globally positive even though certain nuances were expressed in terms of the relevance of the time and energy invested and the limited scope of profile of the recruited participants.

*“The burden could be described as heavy in itself, I had to be insistent, it may have scared some people off, especially doctors … (but it is also about) … fighting against the culture of superficiality … ”*

*Contact person, hospital 3*

3) With regard to **implementation** (i.e. the extent in which an intervention can be fully implemented as planned and proposed), if the process could be fully completed in the three targeted hospitals**,** we notice a quite high proportion of participants who did not complete the questionnaire individually before the round table (*n* = 4, 19%) and/or did not appear at the round table thereafter after doing it (*n* = 7, 32%) and a round table meeting time that did not allow to address all the sub-standard in two hospitals (see discussion).
4) With regard to **practicability** (i.e. the extent to which an intervention can be delivered when resources, time or commitment are constrained in some way)**,** our mode of application is a self-declared questionnaire and the instruction given to the participants was to fill it out intuitively. The individual completion time for the questionnaires was assessed by the respondents present at the round table to be 56.8 min on average (standard deviation 15.4). The number of items per questionnaire left without an answer was 14.8 on average (standard deviation 21), i.e. 10% of the items. The number of ‘Non-applicable’ replies per questionnaire was 5.5 on average (standard deviation 8.7), i.e. 4% of the items. The possibility to leave comments below each item was rarely used overall, and essentially to explain a missing response with “I don’t know” or “to be verified”, less often to justify an answer or to put forward specificities concerning the institution in question. Overall, 27% (6/22) of the participants in the round tables had not completed their questionnaires individually before the meeting and 33% (7/21) of those who completed the questionnaire individually did not appear at the round table. The number of sub-standards that could be discussed during the round table meetings varied between hospitals: 20/20 in the first, 10/20 in the second and 7/20 in the third. This is explained by the fact that the real duration of the meeting, approximately 2 h as planned for the 1st hospital, was unfortunately reduced to 1 h 30 in the second hospital (due to a delay of the participants) and 1 h in the 3rd hospital (due to the integration of the round table in a pre-existing meeting slot).
5) With regard to **integration** (i.e. the level of system change needed to integrate a new program or process into an existing infrastructure), while in the first two hospitals a special date for the round table meeting was agreed upon with the different participants, the example of hospital 3, where the project presentation and then the round table meeting were introduced into the agenda of a pre-existing working group, shows us that our method of applying the V-HLO can be integrated into the normal functioning of an institution. Even though the time available for this meeting was shorter than desired (1 h instead of 2 h), the prioritisation of discussions on the least consensual sub-standards allows these time constraints to be taken into account. Our study benefited from the top management agreement but it is difficult to judge their real commitment to the issue.

*“We have the support of management, but this is not a priority either, we are left to our own devices … ”*

Participant hospital 2

### Strengths and weaknesses regarding health literacy responsiveness

**Fig. 2:**
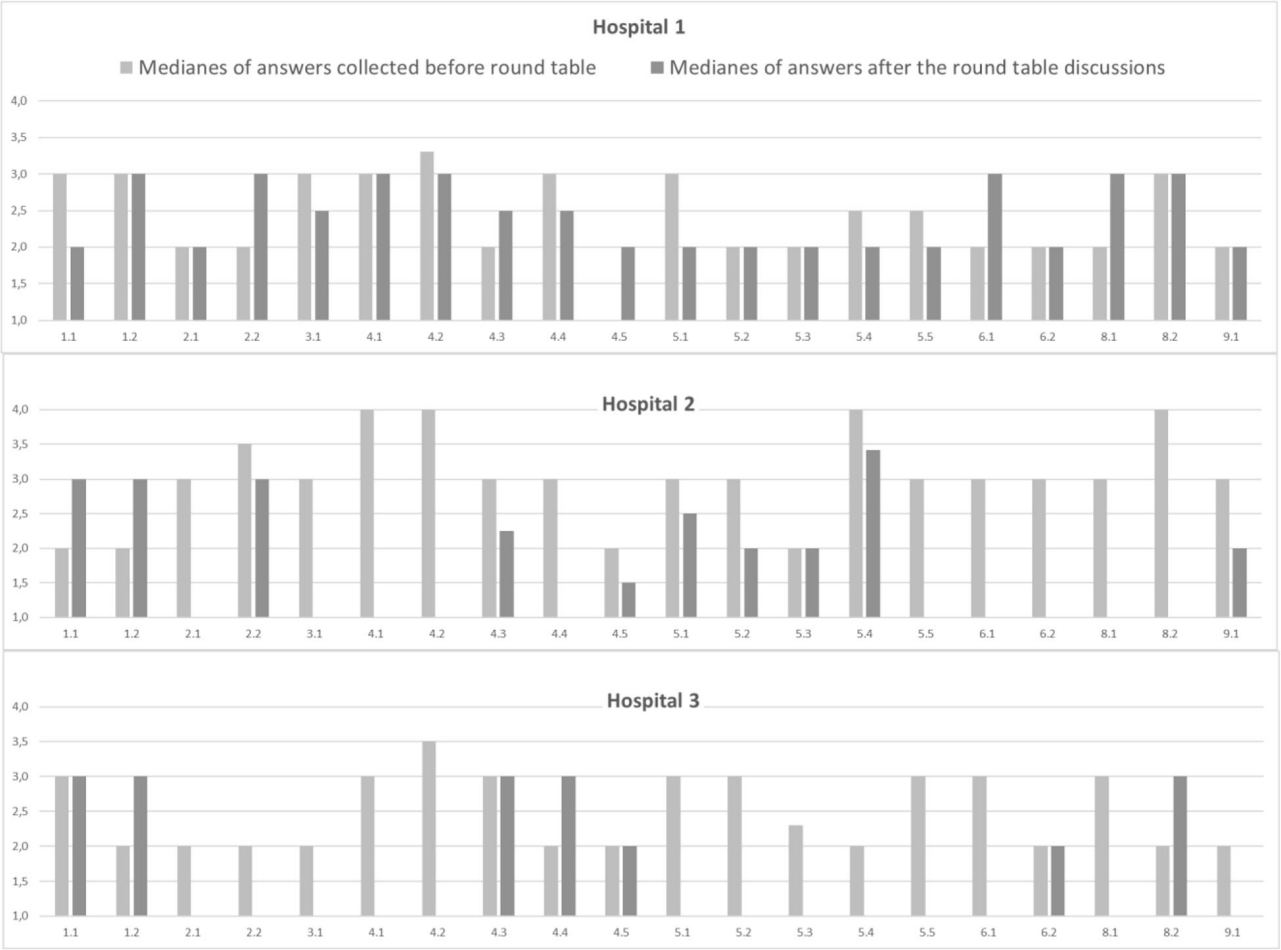
median scores for the different sub-standards of the V-HLO-fr for each hospital. Vertical axis: degree of fulfilment of the sub-standard in the self-assessed hospital: 4 for ‘yes’, 3 for ‘rather yes’, 2 for ‘rather no’ and 1 for ‘no’. Horizontal axis: number of the different sub-standards of the questionnaire

In our study we gained a first insight into the strengths and weaknesses of each of the participating hospitals in terms of health literacy responsiveness. The results of the questionnaires in terms of median scores by sub-standards are given for each hospital in Fig. 2.

The exchange during the round table seems to have influenced the scores attributed both in a positive and negative sense. The strong and weak points identified per hospital are highlighted in Table 3. More weaknesses than strengths were identified. Facilitation of patient navigation to the hospital is overall considered a strong point and the provision of easy-to-read, understand and act-on health information materials a weak point. We observe a lack of discrimination of the tool in hospital 1 at least at the level of the sub-standards for which all numerical values are between 2 and 3.

**Table 3:** Sub-standards of the V-HLO-fr (translated) identified as strengths or weaknesses in terms of health literacy responsiveness per hospital

	Strengths	Weaknesses
**Hospital 1**	None identified	None identified
**Hospital 2**	4.1 The institution allows an easy first contact by internet or telephone. 4.2 The institution supplies all the information necessary to get to the establishment for the purposes of a stay. 5.4 A communication in the mother tongue is made possible thanks to human resources and materials. 8.2 The institution contributes to public health within the bounds of its possibilities.	4.5 Information material on health is made available to patients and visitors.
**Hospital 3**	4.2 The institution supplies all the information necessary to get to the establishment for the purposes of a stay.	2.1 The institution involves the patients in the development and evaluation of the documents and presentation of care. 2.2 The institution involves its personnel in the development and evaluation of the documents and care services. 3.1 The training programmes of the personnel aimed at communicating with patients complies with the requirements of the health literature relative to all communication situations. 4.5 Information material on health is made freely available to patients and visitors. 5.4 A communication in the mother tongue is made possible thanks to human resources and materials. 6.2 The institution supports the patients during the acquisition and development of health literacy responsiveness with a view to developing healthy lifestyles. 9.1 The institution supports the diffusion and development of health literacy responsiveness.

Item outliers within their sub-standards before the meeting, that is to say with a score deviating significantly (more than one point) from the median of their sub-standard, are highlighted in Table 4. These are all potential strengths and weaknesses that were not specifically discussed during the round tables due to the refocusing of discussions at the sub-standard level.

**Table 4:** Item outliers within their sub-standards

	Item outliers toward the top (better score than its sub-standard)	Item outliers toward the bottom (lesser score than its sub-standard)
Hospital 1	4.3.5 The writing spaces are clearly indicated.	3.1.3 Resources are planned for the training of personnel in the standards of health literacy responsiveness. 3.1.6 Internal experts intervene as models, mentors and teachers to promote health literacy responsiveness. 4 .1.7 The contents of the site are available in several languages. 4.2.4 The name of the establishment is clearly indicated outside the building. 4.4.8 The establishment has a guiding system for visually impaired visitors. 5.1.8 The time devoted to interviews with patients is sufficient. 5.4.4 The patients are informed – by a sign, for example – of the possibility to request a translation service.
Hospital 2	1.2.5 Patient enquiries also relate to quality of communication (the clarity of information, for example).	1.2.9 The experience of the patients is solicited (for example, through tracer patients and/or test users – also called ‘mystery patients’) to check how well the patients manage to orient themselves in the establishment. 3.1.1 Health literacy is considered an essential professional skill. Documents attest to this (job offers, job description or staff development plans, for example). 4.1.7 The contents of the site are available in several languages. 5.4.5 All interpreting by minor persons or personnel without specific training is specifically excluded. 5.4.11 All written and audiovisual tools –information leaflets, declarations of consent, etc. – are available in the mother tongues of the main groups of patients.
Hospital 3	None	4.3.4 The new technologies, like touch screens (speaking) or smartphone applications, facilitate orientation within the establishment. 5.3.1 Guidelines for the use and quality of computer applications and new media exist to facilitate communication and the transmission of information. 5.3.4 Computer applications are tested with representatives of the target group before their routine use. 5.3.5 During the use of applications and new media, there is a verification of the patients’ ability to use them. If necessary, relevant training is suggested.

## Discussion

### Main results

The V-HLO-fr tool was fully applied in three hospitals in Liège, Belgium. This constitutes an overall positive signal about its feasibility in the Belgian context. More specifically, the process seems to be acceptable, practicable and integrable. Possible improvements to this process will be discussed below. Our explorative studies also allowed us to provide an overview of the strengths and weaknesses of the participating hospitals in terms of health literacy responsiveness. While the international literature places the emphasis on the efforts to be made in terms of improving navigation [22], the hospitals identify this field as one of their strong points; a similar result was observed in the feasibility study in Vienna. The provision of easy-to-read, understand and act-on health information materials is a field that has been identified as weak. This observation could constitute, in line with the literature [10], a particularly favourable starting point for first improvement projects in the field of health literacy responsiveness. Moreover, this is an area in which the interventions are relatively well described [23] and thus likely to yield assessable results within reasonable deadlines. The four categories of the questionnaire correspond to the self-assessed “degree of fulfilment” of the different items within the institutions, they are ordinal qualitative variables. There is no predefined threshold above or below which a score could be qualified as “strength” or “weakness”. In our study, strengths and weaknesses identified are therefore relative scores and only engage the judgement of the authors on the basis of the graphical representation of the results. It further depends upon the aims of an institution and understanding of the local context, how scores will be interpreted and whether they would be prioritized for action. Taking this into account, an item with a score of 2 could very well be considered as a strength and another with a score of 3 as a weakness. The relative ‘reserve’ expressed by the contact persons during the phone interviews, in contrast with the enthusiasm observed during the round table meetings, may reflect a possible lack of clarity in our communication concerning the objectives of the study: above all, to test a tool and a mode of application. Legitimate but disproportionate expectations in terms of initiation and support for change may have been raised. Even though this study was not designed to accompany let alone evaluate a structural change in the institutions targeted, we still gained a cursory understanding of the impact of the application of the V-HLO-fr as carried out in this study. The telephone interviews with the contact persons in each hospital seem to indicate that the application of the questionnaire and the return of the results to the participants did not result in effective follow-up. However, in hospital 1, the initial collaboration around V-HLO-fr gave rise to the joint development of a ‘guide to the patient as proof-reader’^4^; and in hospital 2, the contact person thinks the study may have indirectly strengthened another project aimed at providing easy-to-read information leaflets for patients.

### Strengths and weaknesses of the study

This study presents strong points. It was conducted in three different institutions that were representative of the diversity of the Belgian hospital landscape, making the results generalizable to other French-speaking hospitals in Belgium.

Our explorative case studies made it possible to field-test the mode of application of the questionnaire, which may inform further studies and feed a discussion about the perspective of its pragmatic use in the field.

This study present limits. A desirability bias in the answers cannot be excluded but is inherent to self-assessments; it is perhaps minimised by the large scope of the questionnaire, the anonymity of votes and the prior assurance of not using the results for benchmarking purposes. Only the experience of the contact persons was explored, and the material constituted by the exchanges during the round tables and telephone interviews was neither recorded nor systematically explored because that went beyond the objectives of explorative case studies. Future feasibility studies should also document the strategy of recruiting the participants undertaken by the contact persons within each institution in order to discuss the differences in the profiles of the participants and the adhesion to the study with regard to the profiles targeted. Likewise, the reasons for the non-completion of the questionnaire or for not participating in the round table should be systematically explored. We also draw attention to the fact that the median scores per sub-standard before and after the round table meetings are the result of different calculations: the first, based on individual responses to the different items of each sub-standard prior to the round table; and the second, based on individual responses during the round table meeting after discussion about sub-standards that were able to be dealt with as a whole. In a general way, the V-HLO was primarily used here as a group facilitation tool and the statistical processing of the results is essentially descriptive.

### Lessons learned about our mode of application

In view of the small number of participants, especially in the two first hospitals, particular attention should be paid to the quality and breadth of the internal network of the contact person chosen. Direct additional recruitment could be envisaged, secondly, to expand the panel of experts. This would become essential for projects looking at pragmatic change, for which it would be important to have the spokespersons for the key stakeholders around the table. For reasons of relevance of the statistics as well as the group dynamic during the round tables, the optimal number of participants targeted would probably be between 9 and 15 [21]. In view of the high proportion of participants who did not complete the questionnaire individually before the round table and/or did not appear at the round table thereafter, an intensification of the follow-up of the participants (e.g. telephone calls, clarification about the methods and the issues of the process or any kind of financial or symbolic compensation) could be considered. The 2 h scheduled for the round table allowed us to address all the sub-standards in Hospital 1; this should be considered as a minimum, taking into account the introductory time and the time required for the group to familiarise themselves with the discussion method. As suggested by Fitch et al., it may be interesting during the round table, when discussing a sub-standard, for the chairman, to also point to items within it frequently left unanswered and/or containing major divergences in inter-individual scores. By drawing attention to later subjects of incomprehension or differences in information between participants, this could constructively feed the discussion and influence the quotations attributed to the sub-standard in question. Our study explores application of the V-HLO-fr and stresses the importance of the self-assessment process in developing organizational health literacy policy and actions. Formalizing this process, e.g. how to recruit, brief and coach participants; how to prepare and facilitate meetings; how to deliberate and decide on scores; how to compile and present results is fundamental in order to improve the quality of the organizational diagnosis as well as to allow for replication of the intervention and possible comparison of results. Similar efforts have been undertaken in other countries, using both the same^5^ and other [24] conceptual framework. Thoroughly integrating the knowledge of these separate projects would further improve this self-assessment process.

### Perspective of use of the V-HLO-fr tool in the field

With regard to more classical forms of structured deliberation, the RAND method would perhaps be more capable of mobilising the participating parties and creating a positive dynamic for change. It would therefore be more suitable for a more pragmatic use, aiming to bring about organisational change, beyond the analytical aim of obtaining an organisational diagnosis. In this perspective, the optimal way of implementing the tool (individual and/or collective, spread over several meetings or not) and the targeted participants will have to be thought through according to the specifics of the context and the overall strategy chosen. Note that this multidisciplinary panel focusing on the completion of the questionnaire could constitute the embryo of a steering committee, which would collectively take on board the results of the organisational diagnosis. Those pragmatic projects would benefit from using a proper conceptual model of change within organisations. For example, in the model of Pichault [25], self-assessment tools have their place in facilitating the emergence of a common formulation of the problems to be resolved, the analysis of internal mobilisation capacities and the enrolment of the different stakeholders.

In our study, the results are presented separately for each hospital. The usefulness of the tools was considered above all as serving the internal dynamics of each institution. A comparison of the hospitals would have been difficult anyway given the heterogeneity of the composition of the expert panels and the different number of sub-standards that had been discussed during the round tables. But, subject to cultural acceptability of the approach, the formalisation of the mode of application of the V-HLO tool could facilitate its use in an approach aimed at comparing performance of hospitals between regions and/or countries (benchmarking) in order to stimulate innovation and transfer of best practices between institutions.

In Belgium, these initiatives should be part of a broader plan to strengthen interest in the issue of health literacy, inspired for example by the findings and proposals of the WHO [26]. The report of the aforementioned Belgian healthcare knowledge centre [16] and the interest of private charitable foundations [27] are encouraging signs.

## Conclusions

Our explorative case study is positive regarding the feasibility of the V-HLO-fr tool. The formalisation of its mode of application, inspired by the RAND method, and the lessons learned about it could inform further studies. The V-HLO-fr tool now has to fully confirm its feasibility in different contexts and find its place in real-world projects to demonstrate its ability to help hospitals to create awareness and formulate targeted actions to improve organizational health literacy and reduce their unnecessary complexity.

## Supplementary Information

**Additional file 1.** Road map for the animation of the round table meeting

**Additional file 2.** Semi-structured phone interview guide

## Data Availability

The datasets used and/or analysed during the current study are available from the corresponding author on reasonable request.

## Abbreviations

V-HLO: Vienna Health Literate Organisation
V-HLO-fr: French version of the Vienna Health Literate Organisation

## Footnotes

1 The ten attributes of Health-Literate Health Care Organizations (Brach C. et al. 2012) [4]:

- Has leadership that makes health literacy integral to its mission, structure, and operations.
- Integrates health literacy into planning, evaluation measures, patient safety, and quality improvement.
- Prepares the workforce to be health literate and monitors progress.
- Includes populations served in the design, implementation, and evaluation of health information and services.
- Meets the needs of populations with a range of health literacy skills while avoiding stigmatisation.
- Uses health literacy strategies in interpersonal communications and confirms understanding at all points of contact.
- Provides easy access to health information and services and navigation assistance.
- Designs and distributes print, audiovisual, and social media content that is easy to understand and act on.
- Addresses health literacy in high-risk situations, including care transitions and communications about medicines.
- Communicates clearly what health plans cover and what individuals will have to pay for services.

2 See: 1. https://www.hphnet.org/knowledge-innovation/#content-working-groups

3 See: https://www.integreo.be/fr/pres-de-chez-vous/liege-et-seraing-relian-reseau-liegeois-integre-pour-une-autonomie-nouvelle

4 See: http://hdl.handle.net/2268/239320

5 See here for an international network to further develop the instruments on measuring organizational health literacy https://m-pohl.net/aims_organizational_health_literacy
