# Supplementary material for: The Vienna self-assessment questionnaire: a usable tool towards more health-literate hospitals? Explorative case studies in three hospitals in Belgium
Source: BMC Health Serv Res. 2021 Mar 31;21:287. doi: 10.1186/s12913-021-06211-y (PMC8011166; doi:10.1186/s12913-021-06211-y)
Supplement: Supplementary file 1 — Additional file 1. Road map for the animation of the round table meeting [file 12913_2021_6211_MOESM1_ESM.pdf]

Roadmap for the round table meeting

(Translated)

• Meeting date fixed via doodle

• Sending of the individual questionnaire by email in electronic form with instructions to return it 2 weeks before the meeting date (with a proposal for technical support in the event of a problem accessing the document).

• Analysis of individual questionnaires

- Reading of any comments left by participants.

- Selection of sub-standards to be discussed as a priority during the round table meeting (ranking through the Mean Absolute Error of the sub-standard, see publication)

• **D-day**: round table (duration = 2 hours)

- Welcome - presentation of investigator / observer and participants (5 min)

- Reminder of the framework in which the meeting takes place and explanation of the objectives of the meeting (5 min, 3 PowerPoint slides)

Framework:

• Investigator thesis within the Primary care and health research unit of ULiège (supported by the Leon Fredericq Funds) - Objective of the thesis (translation-adaptation and feasibility of V-HLO-fr, within an international working group).

• Action within the Rélian project.

• Glossary: “health literacy” and “organizational health literacy”.

Objective of the meeting:

- Achieve a shared health literacy organizational diagnosis of the hospital

- Methodology of the meeting (5 min, 1 dia)

General idea:
• "Strive for consensus", by differentiating "real disagrements" from "artefactual disagrements" (incomprehension vs. lack of information) but without the need for consensus (the final score is the median of the responses of the different participants).
• Necessary "framing" of the discussion for reasons of timing.

Structuration of the discussion:

1) For each sub-standard discussed in plenary (see above): explanation on the sub-standard (reading of the explanatory text of the VHLO-fr questionnaire and, citation of the different criteria to illustrate).

2) Questions for clarification?

3) Displays of the median of the individual responses previously received for a sub-standard, therefore anonymous, numerical score was attributed to each response category of the questionnaire: 4 for 'yes', 3 for 'rather yes', 2 for 'rather no' and 1 for 'no'.

4) Reactions?

5) Anonymous individual scores on paper for the sub-standard discussed.

- Closing round table: individual experience of the round table, with the question *"At the end of this meeting, when I hear" Organizational health literacy, I think…*"

- Acknowledgments and follow-up to be given
