# Supplementary material for: The Vienna self-assessment questionnaire: a usable tool towards more health-literate hospitals? Explorative case studies in three hospitals in Belgium
Source: BMC Health Serv Res. 2021 Mar 31;21:287. doi: 10.1186/s12913-021-06211-y (PMC8011166; doi:10.1186/s12913-021-06211-y)
Supplement: Supplementary file 2 — Additional file 2. Semi-structured phone interview guide [file 12913_2021_6211_MOESM2_ESM.pdf]

# Remote evaluation of the V-HLO-Fr intervention

# Interview guide

(Translated)

| |
|---|
| DATE: |
| IDENTITY/HOSPITAL: |

**Question 1**: How would you qualify the workload requested for the intervention?

More generally: feasibility of the process?

**Question 2**: Did the intervention contribute to your knowledge of the subject "Health literacy in healthcare institutions"?

**Question 3**: Has the round table had, at this stage, an impact in your institution? For example:

**Question 4**: Do you think that the result of organisational diagnosis you have just received could be useful to you?

More globally:

- Utilities / impact of the process?

- Follow-up given / envisaged?

- Any suggestions for improvement?

(Transversely: did context-specific points spontaneously emerge from the discussion: temporality, power relationship, group composition, etc.…?)
